# Concluding Commentary. Life Sciences in an Integrated Medical Curriculum: Continuing the Conversation

**DOI:** 10.15694/mep.2017.000114

**Published:** 2017-06-23

**Authors:** Iain D. Keenan, Barbara A. Jennings

**Affiliations:** 1 Newcastle University; 2 UEA

**Keywords:** Life Sciences, Integrated Curriculum, Anatomy, Student Partnerships

## Abstract

This article was migrated. The article was marked as recommended.

In our opening editorial, we contrasted the time devoted to delivering the Life Sciences curriculum, as a part of medical training, with the limited attention given to it within the medical education literature ( Jennings & Keenan, 2017). In our experience, there are also few opportunities to present and discuss Life Sciences at medical education conferences and perhaps within the wider medical education community. We therefore started this conversation to provide a forum for consideration of the integration and delivery of Life Sciences teaching within medical curricula.

## Life Sciences in an integrated curriculum

Having found only 7 previous publications in MedEdPublish which concerned Life Sciences education before April 2017, we have now been able to more than double that number in the space of three months through this themed issue. We consider this to be a successful achievement and hope that those educators who deliver such topics now feel they have been given more of a voice within the medical education community.

The submissions we received have primarily surrounded innovative learning and teaching approaches including the use of technology with respect to social media ( Whyte & Hennessy, 2017), 3D printing ( Li et al., 2017), peer teaching ( Border et al., 2017; Simpson, Curley, Veloso Costa, & Ahmed, 2017), artistic learning methods ( James, O’Connor, & Nagraj, 2017; Keenan, Hutchinson, & Bell, 2017), the flipped classroom ( Stoner, Caruana, & Stabile, 2017), the impact of psychometric abilities ( Smith et al., 2017) and a description of an integrated Life Sciences course ( Fenoll-Brunet et al., 2017). The theme also includes a call for focus on a particularly concerning worldwide Life Sciences topic ( Pyatt & Bowater, 2017). In addition to original articles, case studies and reviews, suggestions of practical tips for Life Sciences educators have also been included ( Border et al., 2017; Jennings, Bassi, & Loke, 2017; Keenan et al., 2017).

While the majority of the themed articles concerned anatomy education ( Border et al., 2017; James et al., 2017; Keenan et al., 2017; Li et al., 2017; Smith et al., 2017; Stoner et al., 2017; Whyte & Hennessy, 2017), this would perhaps be expected given the apparent proportion of active researchers and published literature in this field when compared to other Life Sciences disciplines. However, we have also received submissions from the relatively less well represented areas of the Life Sciences including physiology ( Simpson et al., 2017), microbiology and public health ( Pyatt & Bowater, 2017), and the Life Sciences more generally ( Fenoll-Brunet et al., 2017; Jennings et al., 2017).

Taken together, the submissions have satisfied all of the themes we proposed on our Editorial, and have started conversations surrounding 1) Learning Gain, Innovation and Teaching Excellence, 2) Research-Led Teaching and Evidence-Based Practice, 3) Integration and Creating a Community of Practice, and 4) Preparing Medical Researchers ( **Table 1**) ( Jennings & Keenan, 2017).

**Table 1.**
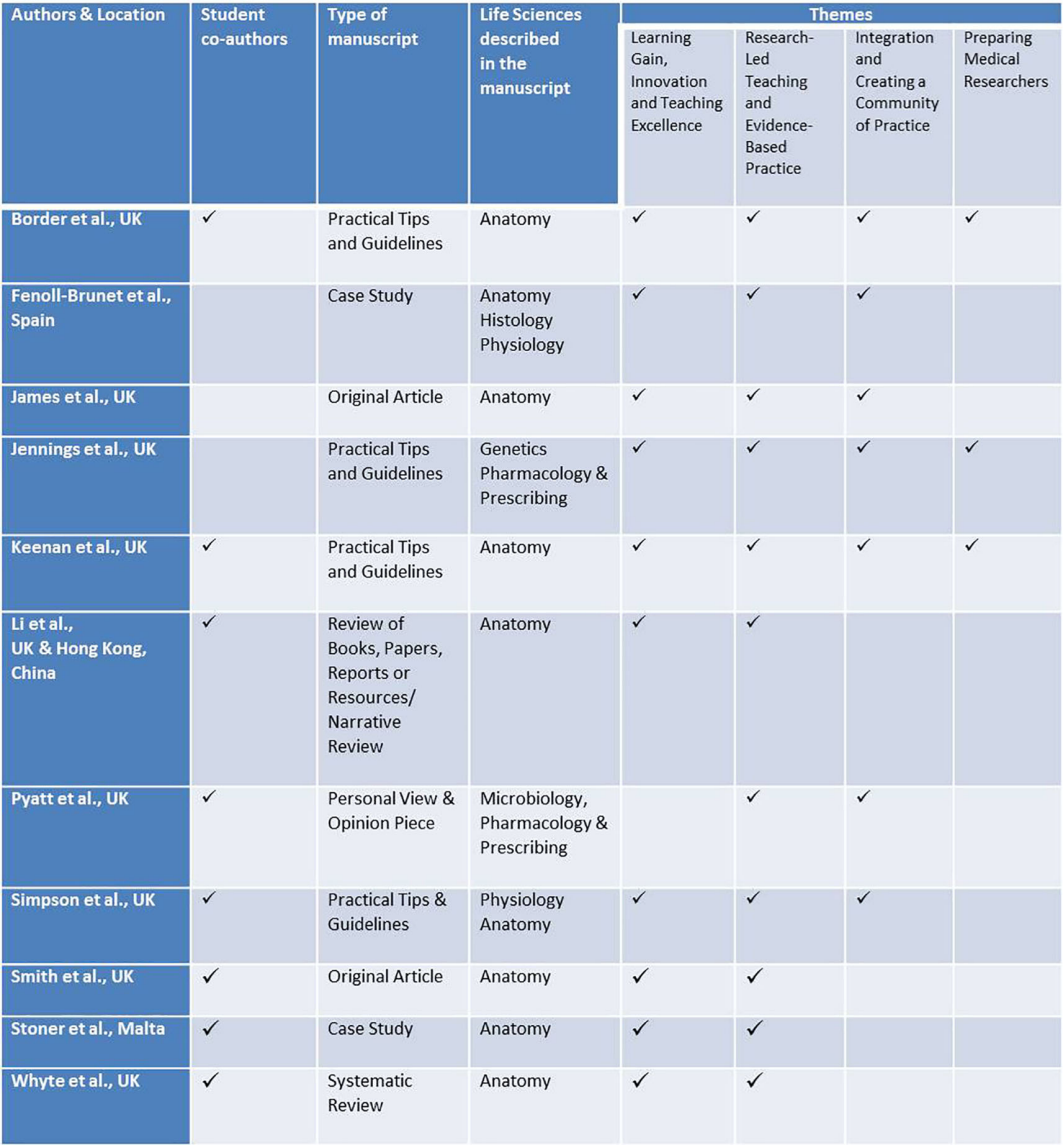
A summary of the disciplines and themes discussed in the papers that comprise this Life Sciences themed edition of the journal. In our opening editorial we encouraged the submission of articles that map to the four themes listed in the final columns of this table. The sciences described in the published articles are indicated with respect to each of these themes. Eight of the eleven manuscripts were co-authored with medical students or recent graduates.

## Synopsis of submissions

The first submission to our theme was a systematic review of social media, originally based within anatomy but extended to undergraduate medical education more widely ( Whyte & Hennessy, 2017). Not only did the authors identify key areas with respect to the use of social media in learning and teaching through implementation of a rigorous review method, they also developed a questionnaire based on these themes in order to provide further insight into this topic during further research. We eagerly await the outcome of this study in a future article. This was a welcome submission not only because of the innovative approach to learning and teaching that was being considered, but it was also valuable from an evidence-based practice point of view and therefore satisfied the first two themes which we outlined in our editorial ( Jennings & Keenan, 2017).

The second themed article concerned practical guidelines for near-peer and peer-peer teaching in anatomy education ( Border et al., 2017) and this time satisfied all of our proposed themes. The recommendations described included important considerations such as curricular integration, teaching strategy and the cultivation of student partnerships and employed a scholarly approach to the theoretical basis and research evidence throughout.

The next submission again provided a review of literature of an innovative approach to Life Sciences teaching involving a recent technology, in this case a narrative review which could form the basis of future research, systematic reviews and perhaps ultimately, wider integration of 3D printing into undergraduate anatomy education ( Li et al., 2017). The topic of innovative anatomy learning methods continued with the next submission, which described life drawing classes for medical students ( James et al., 2017). The authors proposed that not only was this approach suitable for anatomy learning, but also provided more holistic benefits to medical students, an important consideration when integrating novel methods in to curricula.

The theme issue continued with another anatomical paper, this time an original article describing a detailed research study of the impact of psychometric abilities on anatomy learning ( Smith et al., 2017). This work serves to highlight the differences between individual students and their approaches to learning and can provide Life Sciences educators with insights into how their own teaching environments can be adapted to optimise student learning.

The first three submissions all included undergraduate student authors,highlighting contributions to our student partnership theme not only in the work described, but also in the contributions to the work itself. We advocate this approach as such studies can not only embrace the student perspective, but also provide the opportunity for students to develop skills as researchers in medical education. This student partnership trend continued with subsequent submissions, the first of which again aligned with our theme. In this case a description of a call for educational awareness of the global public health problem of antimicrobial resistance ( Pyatt & Bowater, 2017). The authors proposed that this could be achieved through not only appropriate delivery of microbiology teaching in medical curricula, but also through integration with other Life Sciences in addition to clinical sciences.

Student authors were also involved in providing their perspective to the delivery of innovative learning and teaching in the next submission which described practical tips for implementing artistic learning methods in anatomy utilising research-led, evidence-based student partnership approaches for enhancing learning ( Keenan et al., 2017).

While flipped learning is becoming a more established approach, it remains a relatively recent phenomenon which may not yet be widely utilised by Life Sciences educators. A case study of a flipped classroom approach for delivering gastrointestinal anatomy has highlighted a viable alternative to traditional anatomy teaching ( Stoner et al., 2017). While the authors did not observe improvements in student learning versus standard methods, the flipped approach was found to be equally effective and could therefore be used to increase variety and if used appropriately, to encourage engagement and enhance learning of Life Sciences.

The description of the first step towards integration of a novel student-led approach to ultrasound physiology was described in the next submission to the theme ( Simpson et al., 2017) and presented positive self-reported student perceptions. We look forward to future development and evaluation of this work by the authors if and when their approach becomes established, and we hope that consideration of recommendations in the earlier practical guide ( Border et al., 2017) will enable them to do so.

While the initial submissions to the theme considered specific disciplines, the final two articles have provided an appropriate summary of the theme by providing descriptions of work and recommendations for Life Sciences education more generally. It is hoped that all readers will find the recommendations outlined in the practical Life Sciences guide ( Jennings et al., 2017) and the proposal for the implementation of integrated pre-clinical Life Sciences skills ( Fenoll-Brunet et al., 2017) valuable for their practice.

## Organisational culture and future directions

It is worth considering why some Life Sciences are under-represented in this themed issue and in the wider medical literature. It is possible that organisational culture may explain why anatomy remains the dominant discipline. In most medical schools, anatomy departments are organised around the teaching needs of medical students or postgraduate specialists. Therefore, the faculty members may be recruited for a particular interest in education, and their career paths enhanced if they are committed to maintaining a scholarly approach to teaching and learning. There is certainly an established medical education research community and social media community linked to Anatomy. For other science disciplines the pull towards laboratory and translational medical research, or to clinical service laboratories, may leave little time for engagement with, or the development of, discipline-specific educational research. It would be interesting to know more about career pathways that influence the organisation of Life Sciences curricula in Medical Education. This is a topic that has been explored recently for the Clinician Scientist in Germany (Fischer & Fabry, 2016); but we should also consider the career drivers for the non-clinical academic within our medical schools.

Finally we need to acknowledge the need for more rigorous primary research. The articles included in this themed edition demonstrate a faculty and student body that is dynamic and open to the adoption of innovative and disruptive technologies that can enhance learning and teaching. The authorship lists also suggest a community that embraces partnerships between faculty and learners to develop curricula. The research outcomes have included student evaluations and uptake of innovation, which is treated as a marker of success. But the shortage of rigorous primary intervention studies about learning gain; changes in behaviour; and other downstream professional impacts should be considered and rectified.

## Take Home Messages

• There is an enthusiastic educational community, representing a broad range of Life Sciences, keen to share innovative strategies that encourage active learning in medical students.
• Student partnerships are often integral to curriculum development.
• We recognise the need for more rigorous primary research to inform evidence based practice; considering learning gain and the longer term impact of interventions.

## Notes On Contributors

Dr Iain Keenan is a Lecturer in Anatomy within the School of Medical Education at Newcastle University. Iain has a research background in Life Sciences and is currently an academic lead for anatomy teaching and curriculum officer for the undergraduate medical degree programme at Newcastle. Iain also contributes to postgraduate medical education, medical sciences, physician associates and clinical training programmes. Iain is an active investigator in medical education with a particular interest in innovative and creative learning methods in anatomy education and is councillor, Website, Media and Communications Officer and Social Media Editor for the Anatomical Society. Iain is a fellow of the Higher Education Academy. Please see Iain’s twitter account; @dr_keenan

Dr Barbara Jennings joined Norwich Medical School as part of the inaugural team when it was established in 2002, and is a Senior Lecturer in the Medical Education department. She is the academic lead for the genetics curriculum, and for faculty continuous-professional-development. Barbara has a background in cancer research and in clinical molecular diagnostics. Her research spans cancer genetics, genetic epidemiology and pharmacogenetics. Barbara is a member of the MedEdPublish editorial board and is Senior Fellow of the Higher Education Academy. Please see Barbara’s twitter account; @GeneticsMBBS

## Declarations

Iain Keenan and Barbara Jennings are guest Theme Editors for the AMEE MedEdPublish themed issue, Life Sciences in an Integrated Curriculum.
