# Supplementary material for: Concluding Commentary. Life Sciences in an Integrated Medical Curriculum: Continuing the Conversation
Source: MedEdPublish (2016). 2017 Jun 23;6:114. [Version 1] doi: 10.15694/mep.2017.000114 (PMC10885269; doi:10.15694/mep.2017.000114)
Supplement: Peer review history

# Reviewer report: Reviewer response for version 1

**DOI:** 10.21956/mep.19274.r28024

**Published:** 2019-09-30

**Authors:** Ken Masters

**Affiliations:** 1 Sultan Qaboos University

This review has been migrated. The reviewer awarded 5 stars out of 5 This is a very nice, coherent and succinct wrap-up of the articles submitted to the special themed issue. Table 1 provides a useful overview at a glance. The value of this themed issue is enhanced by the wide variety of subjects and paper types that have been produced, from Literature Reviews to Case Studies. It is also encouraging to see the number of student co-authors on the papers submitted.Overall, a very valuable and interesting theme.

Reviewer Expertise:

NA

No decision status is available

# Reviewer report: Reviewer response for version 1

**DOI:** 10.21956/mep.19274.r28023

**Published:** 2017-06-23

**Authors:** Trevor Gibbs

**Affiliations:** 1 AMEE

This review has been migrated. The reviewer awarded 5 stars out of 5 Thanks you Barbara and Iain for a very thorough synopsis of the themed papers,it makes for very good reading and certainly asks more questions for further discussion. It was certainly very healthy to note the contributions to this theme from the students- that needs more encouragement. I would agree with you that we need to bring more basic and life science teachers into the discussion arena and encourage more research from their specific areas.Although from your synopsis table you have ticked seven of the papers as contributing to integration and community of practice, I personally thought that this was an area that needs improvement, in its methodology and eventual research- we still seem to be seeing stand alone activities in the life sciences despite the talk of increased integration and relevance.Many thanks for co-editing this theme

Reviewer Expertise:

NA

No decision status is available
